# Severe Rhabdomyolysis and Acute Kidney Injury Following Rosuvastatin Reinitiation During Ticagrelor Therapy: A Case Report and Literature Review

**DOI:** 10.7759/cureus.117125

**Published:** 2026-09-29

**Authors:** Shahana Alasgarli, Emil Gasimov, Taleh Naghdaliyev, Gulay Mammadova, Anar Karimkhanov

**Affiliations:** 1 Cardiology, Yeni Klinika, Baku, AZE; 2 Intensive Care Unit, Yeni Klinika, Baku, AZE; 3 Nephrology, Yeni Klinika, Baku, AZE

**Keywords:** acute kidney injury, creatine kinase, rhabdomyolysis, rosuvastatin-induced rhabdomyolysis, ticagrelor-induced rhabdomyolysis

## Abstract

Rhabdomyolysis is a potentially life-threatening condition characterized by skeletal muscle injury, marked elevation of creatine kinase (CK), and potential development of acute kidney injury (AKI). Statins are a well-recognized cause of rhabdomyolysis, although the risk may be influenced by concomitant medications and patient-related factors. We present a case of severe rhabdomyolysis and AKI following initiation of high-intensity rosuvastatin therapy after percutaneous coronary intervention.

A 66-year-old woman with type 2 diabetes mellitus, hypertension, and hyperlipidemia was admitted with acute coronary syndrome. Coronary angiography revealed a critical lesion in the left anterior descending artery, and a drug-eluting stent was implanted. Her low-density lipoprotein cholesterol was 184 mg/dL, and her triglycerides were 356 mg/dL. She was discharged on rosuvastatin 40 mg once daily, ticagrelor 90 mg twice daily, aspirin 75 mg once daily, perindopril, and amlodipine. One month later, she developed generalized weakness, fatigue, and severe myalgia. Initial laboratory investigations demonstrated elevated serum creatinine, alanine aminotransferase, and aspartate aminotransferase. Two days later, she developed anuria, severe muscle pain, and dark-red urine. On admission, CK and serum creatinine were markedly elevated. She denied other medication use, toxic exposure, or recent muscle trauma. Rhabdomyolysis with severe AKI was suspected, and she was admitted to the intensive care unit. Rosuvastatin was immediately discontinued, and ticagrelor was replaced with clopidogrel. Despite intravenous hydration and supportive treatment, metabolic acidosis and renal dysfunction worsened, with a serum creatinine level of 6.3 mg/dL, BUN of 82 mg/dL, and bicarbonate of 15 mmol/L. Hemodialysis was initiated. On the fourth day, she developed severe dyspnea and hypoxemia associated with pulmonary congestion, requiring additional consecutive hemodialysis sessions. Cardiac causes of pulmonary edema were excluded by electrocardiography and echocardiography. Her clinical condition subsequently stabilized, and CK and creatinine levels progressively decreased. She was discharged with continued renal monitoring, and serum creatinine decreased to 1.3 mg/dL by the third week after discharge.

This case highlights the potential for severe rhabdomyolysis and AKI following high-intensity rosuvastatin therapy, particularly in patients with multiple comorbidities. Early recognition of myalgia, dark urine, oliguria, and markedly elevated CK levels is essential. Prompt discontinuation of the suspected medication, aggressive supportive management, and timely initiation of renal replacement therapy can be crucial in preventing further complications and facilitating recovery.

## Introduction

Statins are the cornerstone of lipid-lowering therapy and play a central role in preventing cardiovascular morbidity and mortality. Although generally well tolerated, statin-associated muscle symptoms represent an important adverse effect, ranging from mild myalgia to the rare but potentially life-threatening complication of rhabdomyolysis [1]. Statin-associated rhabdomyolysis involves severe muscle injury with marked creatine kinase (CK) elevation. It may be complicated by acute kidney injury (AKI), electrolyte abnormalities, and, in severe cases, the need for renal replacement therapy [1,2]. Rhabdomyolysis is characterized by marked CK elevation and may be accompanied by renal impairment and/or myoglobinuria [1,2]. The incidence of rhabdomyolysis has been estimated at 13 cases per 100,000 patient-years [3].

The risk of statin-induced rhabdomyolysis is influenced by several factors, including high statin exposure, advanced age, renal or hepatic dysfunction, comorbidities, and concomitant medications that may alter statin metabolism or transport [4,5]. Drug-drug interactions are particularly relevant in patients receiving multiple cardiovascular medications, as increased systemic exposure to statins may substantially increase the risk of severe muscle toxicity. Reinitiation of a statin after previous exposure may also present a clinical challenge, particularly when additional interacting medications have been introduced.

We present a case of severe rhabdomyolysis with dialysis-requiring AKI following reinitiation of rosuvastatin in a patient receiving concomitant ticagrelor therapy. The case highlights the importance of recognizing severe statin-associated muscle toxicity, considering potential drug-drug interactions, and maintaining a high index of suspicion when patients develop muscle injury and markedly elevated CK levels during combination cardiovascular therapy [6].

## Case presentation

A 66-year-old woman had undergone coronary angiography three years earlier for stable angina, which revealed non-critical coronary artery stenoses. Her medical history was significant for type 2 diabetes mellitus, hypertension, and hyperlipidemia. Following angiography, she was prescribed rosuvastatin 20 mg once daily. The patient remained asymptomatic during the first three months of treatment. However, after three months, she discontinued rosuvastatin and aspirin on her own initiative while continuing her antihypertensive therapy. Three years later, she presented to the emergency department with an acute coronary syndrome. Following clinical evaluation, coronary angiography was performed and revealed a critical lesion in the left anterior descending (LAD) coronary artery. A drug-eluting stent was subsequently implanted in the LAD. At that time, laboratory investigations demonstrated an elevated low-density lipoprotein cholesterol (LDL-C) level of 184 mg/dL and a triglyceride (TG) level of 356 mg/dL. During her hospital stay, no significant abnormalities were detected in the biochemical or complete blood count analyses. She continued perindopril 10 mg once daily and amlodipine 10 mg once daily. Ticagrelor 90 mg twice daily, rosuvastatin 40 mg once daily, and aspirin 75 mg once daily were initiated. The patient was discharged home with this medication regimen.

One month after discharge, the patient presented to the emergency department with generalized weakness, fatigue, and myalgia. An electrocardiogram was performed, and laboratory investigations were obtained. Serum creatinine was 1.7 mg/dL, alanine aminotransferase (ALT) was 78 U/L, and aspartate aminotransferase (AST) was 94 U/L. The C-reactive protein (CRP) level and complete blood count were within normal limits. The patient was advised to follow up with a nephrologist and a gastroenterologist as an outpatient.

Two days later, the patient's son contacted her cardiologist and reported that the patient had developed anuria at home accompanied by severe muscle pain. She was immediately advised to present to the hospital for further evaluation. Upon admission, her blood pressure was 165/78 mmHg, heart rate was 96 beats per minute, and oxygen saturation was 93% on room air. She reported that over the preceding two days, her urine had become dark red and had markedly decreased in volume. She denied taking any other medications, exposure to toxic substances, or recent muscle trauma. A comprehensive laboratory evaluation was performed. Laboratory investigations revealed markedly elevated total CK and serum creatinine levels (Table 1).

**Table 1:** Comprehensive laboratory evaluation on admission

Laboratory test	Results	Reference value
Hb (hemoglobin)	10.9 g/dL	12.5-15.1 g/dL
Hct (hematocrit)	31%	36-47%
PLT (platelet count)	264 × 10^9/L	184-433 × 10^9/L
WBC (white blood cell count)	12.56 × 10^9/L	5.2-9.2 × 10^9/L
Creatinine (serum creatinine)	5.47 mg/dL	0.5-0.9 mg/dL
Potassium (K+, serum potassium)	4.8 mmol/L	3.5-5.1 mmol/L
Sodium (Na+, serum sodium)	143 mmol/L	135-145 mmol/L
Urea (serum urea)	159 mg/dL	15-40 mg/dL
BUN (blood urea nitrogen)	74 mg/dL	8-20 mg/dL
NT-proBNP	56 pg/mL	<125 pg/mL
Troponin I	0.1 ng/mL	<0.100 ng/mL
CRP (C-reactive protein)	47.8 mg/L	<5 mg/L
AST (aspartate aminotransferase)	744 U/L	10-35 U/L
ALT (alanine aminotransferase)	379 U/L	7-35 U/L
Total CK (creatine kinase)	53,427 U/L	26-192 U/L

The presence of severe, persistent myalgia, a history of ticagrelor and rosuvastatin use, and markedly elevated serum creatinine and total CK levels raised suspicion for rhabdomyolysis, and the patient was admitted to the intensive care unit. A chest X-ray obtained after admission to the intensive care unit revealed no significant abnormalities (Figure 1).

**Figure 1:**
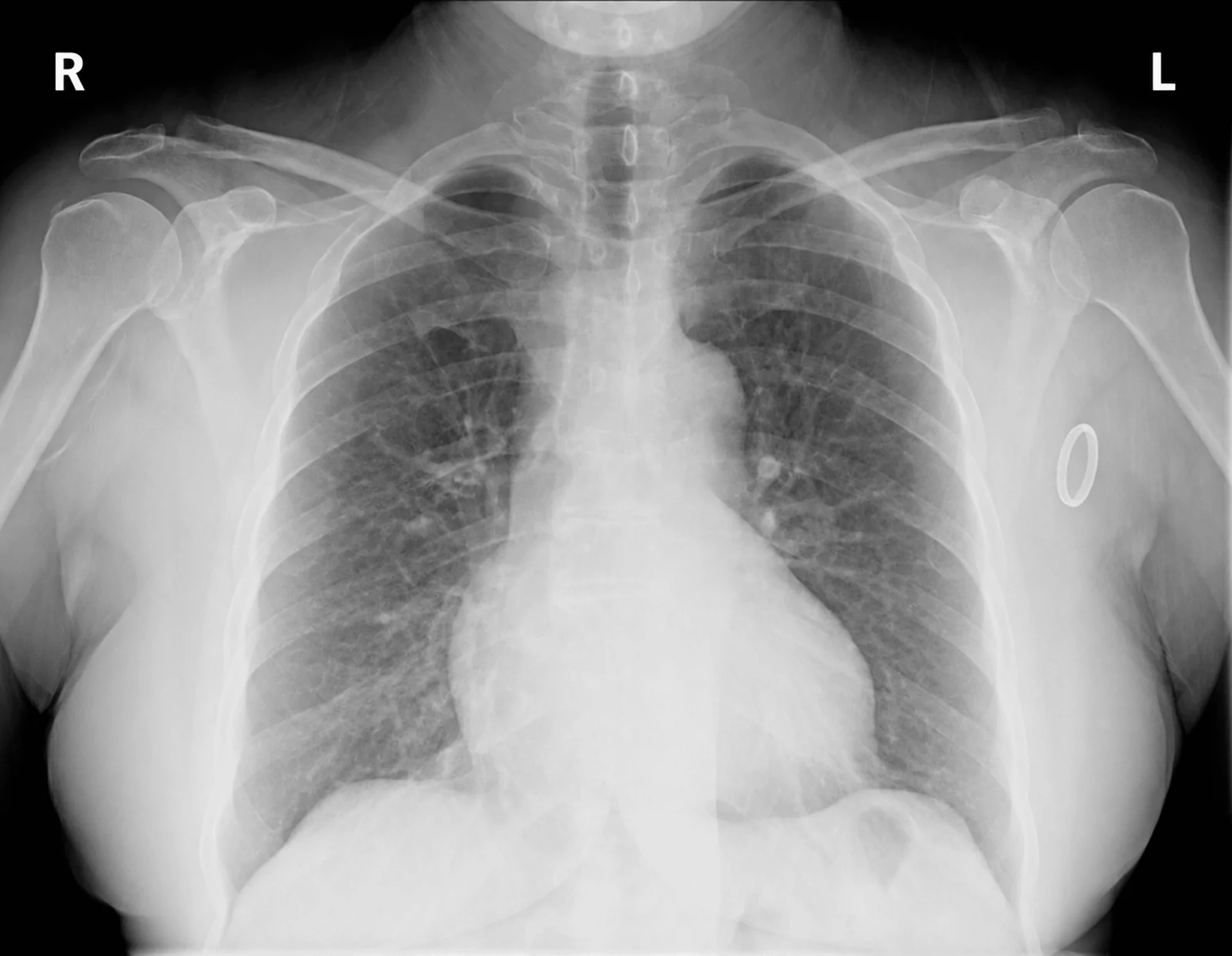
Chest X-ray on admission showing no focal pulmonary opacity, pulmonary edema, pleural effusion, or pneumothorax

Upon admission to the intensive care unit, rosuvastatin was discontinued, and ticagrelor was replaced with clopidogrel 75 mg once daily because of suspicion of a drug interaction between ticagrelor and rosuvastatin. On the first day, intravenous hydration was initiated, along with measures to correct the acidosis. The patient was oliguric.

The following day, the patient developed worsening metabolic acidosis, with a pH of 7.27, lactate of 1.3 mmol/L, bicarbonate of 15 mmol/L, and base excess of −13.3 mmol/L. Daily urine output was 155 mL/day. Renal function further deteriorated, with a serum creatinine level of 6.3 mg/dL, BUN of 82 mg/dL, urea of 185 mg/dL, and serum potassium level of 6.6 mmol/L. The patient remained oliguric. Given the worsening metabolic acidosis, severe AKI, and oliguria, hemodialysis was initiated, and the first session was performed.

Over the following two days, the patient did not undergo further dialysis. However, on the fourth day, she developed severe dyspnea, with oxygen saturation decreasing to 85%. A chest X-ray revealed pulmonary congestion (Figure 2).

**Figure 2:**
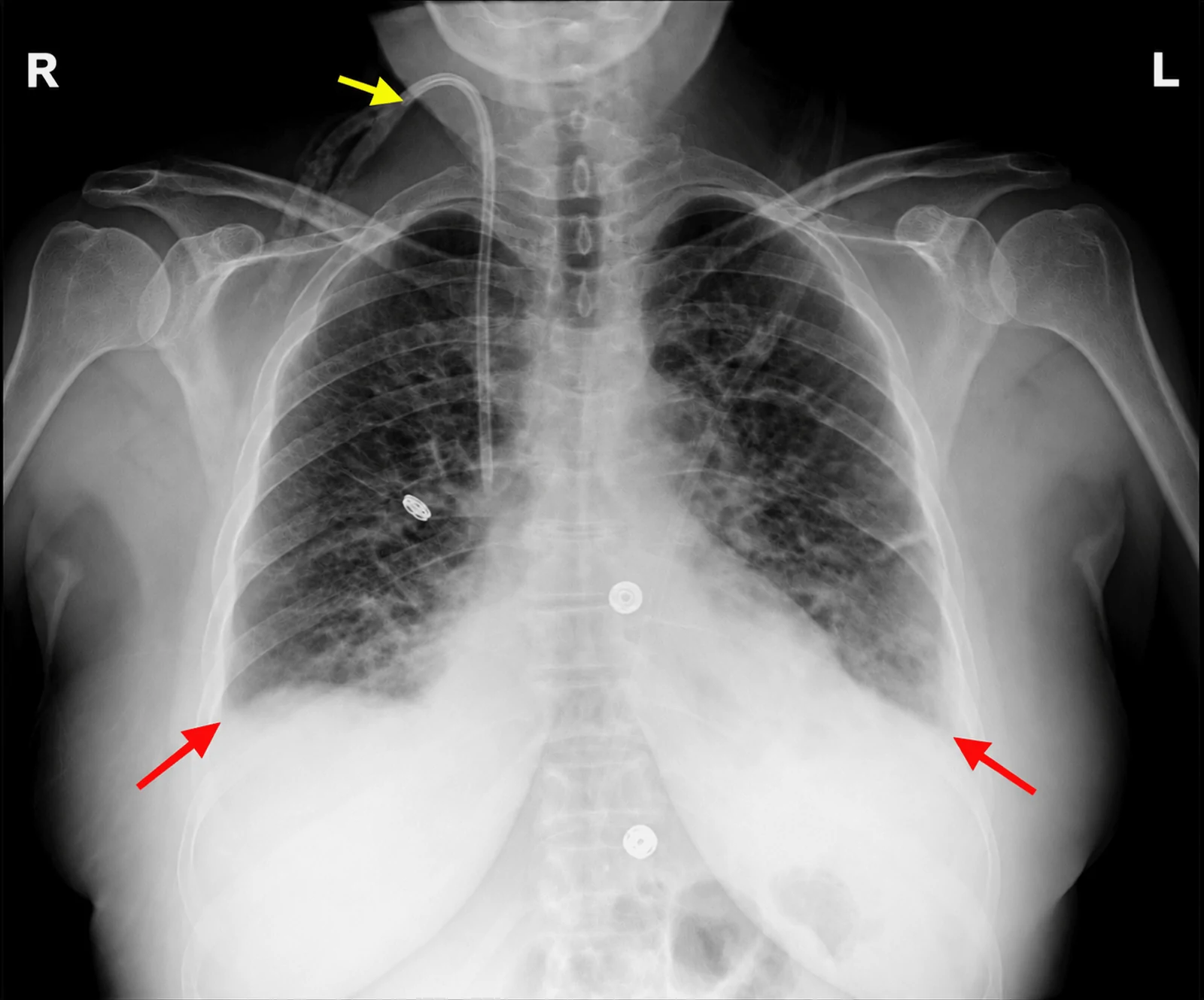
Chest X-ray showing the dialysis catheter (yellow arrow) and possible pleural effusions (red arrows)

A cardiac cause of pulmonary edema was excluded by electrocardiography and echocardiography. The patient subsequently underwent consecutive hemodialysis sessions over the following two days.

After several days, the patient's condition stabilized, and she was transferred to the general ward. Over the following days, complete blood count, electrolyte levels, serum creatinine, and total CK levels were monitored regularly, with a gradual decline in both creatinine and total CK levels (Figure 3).

**Figure 3:**
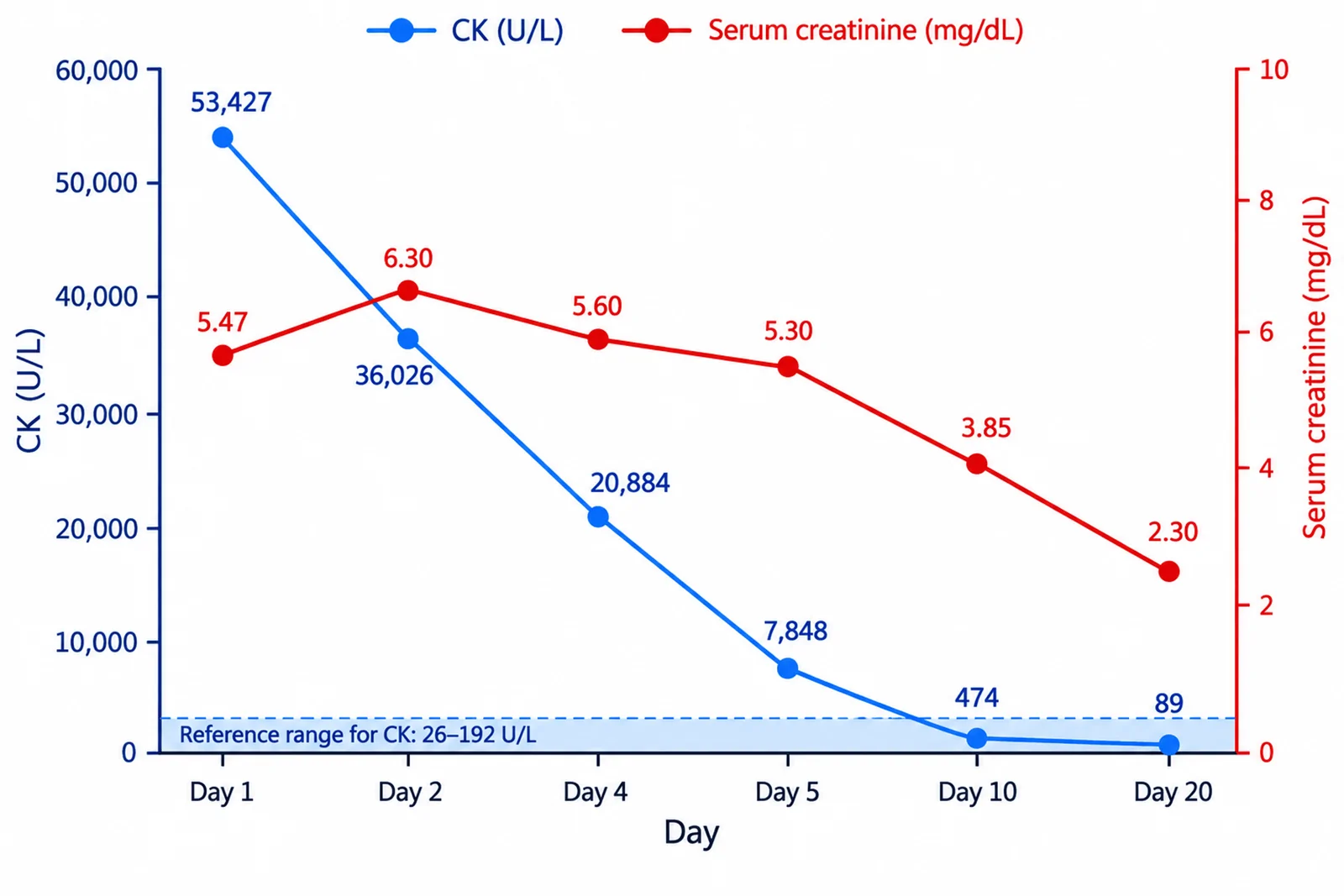
Serial changes in total CK and serum creatinine levels during hospitalization. Both parameters showed a progressive decline following treatment Reference ranges are 26-192 U/L for total CK and 0.5-0.9 mg/dL for serum creatinine. CK: creatine kinase

The patient was discharged home, and serum creatinine levels were monitored weekly. By the end of the third week after discharge, the serum creatinine level had decreased to 1.3 mg/dL. At the most recent follow-up, the patient was receiving clopidogrel 75 mg once daily, aspirin 75 mg once daily, perindopril 10 mg once daily, and amlodipine 10 mg once daily.

## Discussion

Statin-associated rhabdomyolysis is a rare but potentially life-threatening complication of statin therapy. It is characterized by severe skeletal muscle injury with marked elevation of CK and may be accompanied by myoglobinuria and AKI. The risk of statin-related muscle toxicity increases with higher systemic statin exposure. It may be amplified by drug-drug interactions, particularly when concomitant medications interfere with statin transport or metabolism [1-4]. In the present case, the development of severe myalgia, dark-red urine, profound CK elevation, and rapidly progressive AKI approximately one month after reinitiation of high-dose rosuvastatin in combination with ticagrelor is highly suggestive of severe statin-associated rhabdomyolysis [5].

A key feature of this case is the patient's prior tolerance of rosuvastatin. The patient had previously received rosuvastatin 20 mg daily for several months without evidence of muscle toxicity. However, following the acute coronary event, rosuvastatin was reintroduced at a higher dose of 40 mg daily together with ticagrelor 90 mg twice daily. This temporal association raises the possibility that increased rosuvastatin exposure from concomitant ticagrelor use contributed to rhabdomyolysis. Previous reports have described rhabdomyolysis occurring during concomitant ticagrelor and rosuvastatin therapy, supporting a clinically relevant drug-drug interaction [6,7].

The pharmacokinetic mechanism of this interaction has recently been investigated. Rosuvastatin is a substrate of several membrane transporters, including breast cancer resistance protein (BCRP; ABCG2), which limits its intestinal absorption by promoting efflux back into the intestinal lumen. Ticagrelor inhibits BCRP-mediated transport. In vitro and pharmacokinetic modeling studies suggested that this interaction could increase systemic rosuvastatin exposure. Importantly, a subsequent randomized crossover study in healthy volunteers demonstrated that ticagrelor increased rosuvastatin area under the concentration-time curve and maximum plasma concentration by approximately 2.6-fold and prolonged its half-life from 3.1 to 6.6 hours. These findings support intestinal BCRP inhibition as the most plausible mechanism underlying the clinically relevant interaction between the two drugs [8,9]. Although ticagrelor also inhibits OATP1B1, OATP1B3, and OATP2B1 in vitro, available clinical evidence suggests that inhibition of intestinal BCRP, rather than hepatic OATP-mediated uptake, is the predominant mechanism responsible for the increase in rosuvastatin exposure [3].

Increased systemic exposure to rosuvastatin may enhance its intrinsic myotoxic effects. The exact molecular mechanisms of statin-associated muscle injury are not completely established. However, proposed mechanisms include impaired mitochondrial function, reduced synthesis of mevalonate pathway products such as coenzyme Q10, altered muscle-cell membrane integrity, and increased susceptibility to muscle injury. At sufficiently high intracellular or systemic statin exposure, these mechanisms may cause skeletal muscle necrosis, resulting in the release of CK and myoglobin into the circulation. In severe rhabdomyolysis, myoglobin can contribute to renal injury through tubular obstruction, oxidative injury, and direct tubular cytotoxicity, while volume depletion and metabolic disturbances may further aggravate renal dysfunction [9].

The renal presentation in this case is particularly important because contrast-associated AKI (CA-AKI) represents an alternative explanation for AKI in patients undergoing coronary angiography or percutaneous coronary intervention (PCI). CA-AKI is generally defined by an increase in serum creatinine occurring within approximately 48-72 hours after intravascular contrast exposure, although some definitions extend the observation period to several days [10,11]. In contrast, the renal deterioration in our patient developed approximately one month after coronary angiography and stent implantation, making a direct temporal relationship with contrast exposure unlikely. Furthermore, the renal impairment occurred in the setting of severe myalgia, dark-red urine, and extreme CK elevation, providing a clear alternative mechanism for AKI. The clinical picture was therefore more consistent with pigment nephropathy secondary to rhabdomyolysis than with CA-AKI.

This distinction is clinically relevant because AKI following coronary intervention should not automatically be attributed to contrast exposure. Patients undergoing PCI may have multiple simultaneous risk factors for AKI, including hemodynamic instability, heart failure, diabetes, pre-existing renal dysfunction, nephrotoxic medications, and muscle injury [11]. Current kidney disease guidance emphasizes that patients who develop renal dysfunction after contrast exposure should be evaluated for other potential causes of AKI rather than assuming a causal relationship with contrast media [11]. In the present case, the delayed onset of renal dysfunction, marked CK elevation, dark urine consistent with myoglobinuria, and subsequent renal recovery after withdrawal of the suspected medications collectively support rhabdomyolysis-associated AKI [12].

The clinical course further supports the suspected drug-related mechanism. After discontinuing rosuvastatin and replacing ticagrelor with clopidogrel, CK levels progressively declined from a peak of 53,427 U/L to 89 U/L by day 20. Renal function also gradually recovered, with serum creatinine decreasing to 1.3 mg/dL by the third week after discharge. The temporal relationship between withdrawal of the suspected interacting drugs, progressive resolution of muscle injury, and subsequent renal recovery provides additional support for a drug-associated mechanism. Nevertheless, because this is a single case, a definitive causal relationship between ticagrelor and rhabdomyolysis cannot be established, and the interaction should be regarded as a probable contributing factor rather than an independently proven cause.

Figure 4 illustrates the proposed pharmacokinetic and clinical pathway linking ticagrelor to increased rosuvastatin exposure through intestinal BCRP inhibition, followed by skeletal muscle injury, myoglobin release, and AKI. The figure is particularly relevant to this case because it integrates the emerging pharmacokinetic evidence with the clinical sequence observed in our patient.

**Figure 4:**
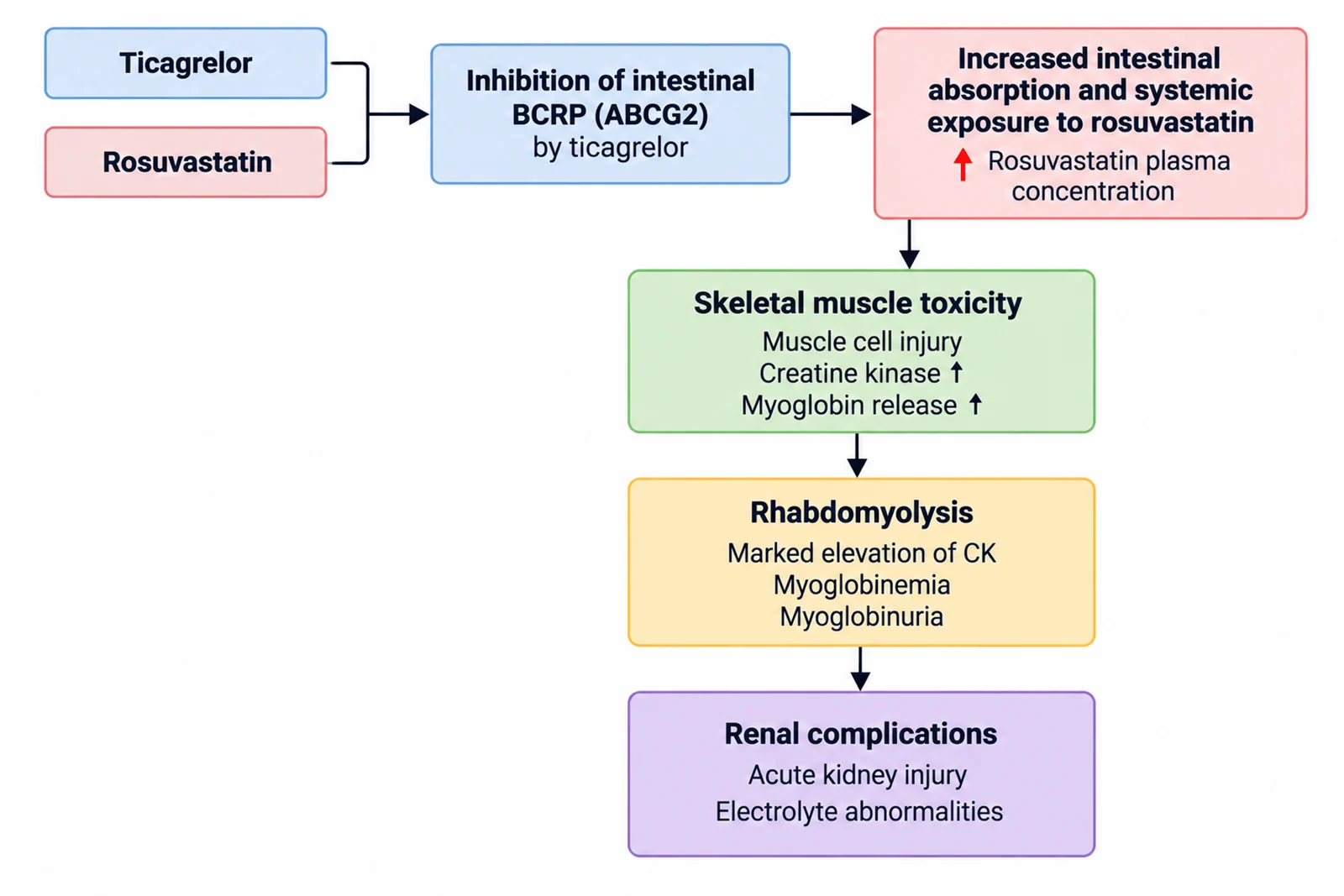
Proposed mechanism of the ticagrelor–rosuvastatin drug interaction leading to rhabdomyolysis and AKI. Ticagrelor inhibits the intestinal breast cancer resistance protein (BCRP/ABCG2), resulting in increased intestinal absorption and systemic exposure to rosuvastatin. The subsequent increase in rosuvastatin exposure may enhance skeletal muscle toxicity, leading to muscle cell injury, marked elevation of CK, and myoglobin release. Myoglobinuria and renal injury may subsequently develop, potentially resulting in severe rhabdomyolysis with AKI The figure was created in PowerPoint 2016 (Microsoft Corp., Redmond, WA, USA) based on the mechanism described in [8]. AKI: acute kidney injury, CK: creatine kinase

Although rhabdomyolysis associated with statin therapy is uncommon, several case reports have described severe muscle injury and AKI in patients receiving concomitant ticagrelor and rosuvastatin. A literature search was conducted using PubMed/MEDLINE and Google Scholar to identify previously reported cases of rosuvastatin-associated rhabdomyolysis, with particular attention to cases involving concomitant ticagrelor therapy. The reported cases vary in terms of rosuvastatin dose, time to onset, severity of CK elevation, and degree of renal involvement. Several patients developed severe AKI requiring renal replacement therapy, suggesting that this drug combination may be clinically relevant in susceptible individuals. Importantly, the reported onset of rhabdomyolysis has ranged from several weeks to several months after initiation of concomitant therapy, making temporal recognition of this adverse drug reaction challenging. Table 2 summarizes selected previously reported cases and compares them with the present case [7-9,12].

**Table 2:** Comparison of previously reported cases of rhabdomyolysis associated with concomitant ticagrelor and rosuvastatin therapy and the present case. The table summarizes patient characteristics, rosuvastatin dose, time to onset, peak CK levels, AKI, requirement for renal replacement therapy, and clinical outcomes AKI: acute kidney injury, CK: creatine kinase, MI: myocardial infarction, NR: not reported, NSTEMI: non-ST-elevation myocardial infarction, PCI: percutaneous coronary intervention, ST: ST segment

Study	Patient characteristics	Rosuvastatin dose	Time to rhabdomyolysis	Peak CK	AKI	Renal replacement therapy	Outcome
Kostapanos et al., 2008 [7]	Patient with acute coronary syndrome/PCI receiving ticagrelor and rosuvastatin	Not clearly reported	After initiation of ticagrelor	NR	Yes	Not reported	Renal function deteriorated; rhabdomyolysis attributed to increased rosuvastatin exposure
Samuel et al., 2017 [12]	Post-MI patient receiving high-intensity rosuvastatin and ticagrelor	High-intensity dose	~1 month	338,601 IU/L	Yes	Hemodialysis (4 sessions)	Clinical and renal improvement after withdrawal of both drugs
Sibley et al., 2021 [6]	74-year-old man after non-ST-elevation myocardial infarction and drug-eluting stent implantation	40 mg/day	~1 month	NR	Yes	Not clearly reported	Improved after discontinuation
Lehtisalo et al., 2023 - patient A [8]	Older man after ST-elevation myocardial infarction/PCI	20-40 mg/day	~1-2 months	NR	Yes	Dialysis	Renal function recovered after discontinuation
Lehtisalo et al., 2023 - patient B [8]	Woman in her 80s after unstable angina	20-40 mg/day	~1 month	NR	No significant AKI	No	Recovered after rosuvastatin discontinuation
Lehtisalo et al., 2023 - patient C [8]	Woman in her 60-70s after NSTEMI/PCI	20-40 mg/day	~6 months	NR	Severe AKI	No	Renal function recovered after discontinuation
Present case	Patient receiving rosuvastatin and ticagrelor after coronary intervention	40 mg/day	~1 month	53,427 U/L	Yes	Yes	CK normalized to 89 U/L by day 20; renal function subsequently improved

Overall, this case highlights a potentially underrecognized interaction between ticagrelor and high-dose rosuvastatin. Although both drugs are frequently prescribed after acute coronary syndromes and PCI, clinicians should be aware that ticagrelor may substantially increase rosuvastatin exposure, potentially increasing the risk of severe muscle toxicity. The development of unexplained myalgia, dark urine, or renal dysfunction in patients receiving this combination should prompt early measurement of CK and renal function and consideration of drug-related rhabdomyolysis. Early recognition and withdrawal of the suspected offending agents may prevent progression to severe AKI and the need for prolonged renal replacement therapy.

## Conclusions

Ticagrelor and rosuvastatin are commonly used together in patients with acute coronary syndromes; however, their concomitant use may increase the risk of severe statin-associated muscle toxicity. Ticagrelor-mediated inhibition of intestinal BCRP can substantially increase systemic exposure to rosuvastatin and may contribute to severe rhabdomyolysis and AKI. In patients developing AKI after coronary intervention, rhabdomyolysis should be considered as a potential cause of renal dysfunction, particularly when accompanied by myalgia, dark urine, or marked elevation of CK. Early recognition of this potential drug interaction, prompt measurement of CK and renal function, and discontinuation or modification of the suspected medications may prevent progression to severe renal injury and reduce the need for renal replacement therapy.
